# Supplementary material for: Effects of cyproheptadine on body weight gain in children with nonorganic failure to thrive in Taiwan: A hospital-based retrospective study
Source: PLoS One. 2021 Oct 19;16(10):e0258731. doi: 10.1371/journal.pone.0258731 (PMC8525746; doi:10.1371/journal.pone.0258731)
Supplement: S1 Table — (PDF) [file pone.0258731.s001.pdf]

S1 Table. Cyproheptadine use in underweight and/or malnourished children

Reference # (year)	Study design	Age (years)	Sex	Sample size (control/ case	CH dose (mg/kg/day)	CH duration (months)	Mean weight gain (g/day)	Height velocity	Outcome
11 (2009)	RCT	2–10 years	Both	21 (11/10)	0.1 HS	4	11.66	Improvement in cases but not controls	Greater weight gain (0.7 vs. 0.1 kg) and growth (1.7 vs. 0.8 cm) at 2 months and increased appetite and IGF-1levels in the CH vs. placebo group
19 (2010)	RCT	6–15 years	Both	70 (33/37)	0.3 TID	2	22.32	NA	Significant weight gain in a CH-treated child
18 (2014)	RCT	2–5 years	Both	77 (40/47)	0.25 BID	1	20.00	No improvement in cases or controls	Higher weight in the CH vs. placebo group at 1 month (0.6 vs. 0.1 kg); no difference in weight or height at 2 months
7 (2014)	RS cohort	7 months–6 years	Both	127 (45/82)	0.25 BID	Unclear	NA	NA	Improvement in weight[b] and mealtime and feeding behaviors; CH, well tolerated overall
Our study (2020)	RS	3–11 years (girls: 3–10; boys: 3–11) prepubertal stage	Both	788 (733/50)	0.3 mg TID	3.24 (97.22 days, 14–532)	26.45	Improvement in cases	Higher weight gain in the CH group

RCT, randomized control study; RS, retrospective; NA, not applicable; IGF-1, insulin-like growth factor
