# Supplementary material for: Effects of cyproheptadine on body weight gain in children with nonorganic failure to thrive in Taiwan: A hospital-based retrospective study
Source: PLoS One. 2021 Oct 19;16(10):e0258731. doi: 10.1371/journal.pone.0258731 (PMC8525746; doi:10.1371/journal.pone.0258731)
Supplement: S1 Fig — (PDF) [file pone.0258731.s002.pdf]

S2 Figure. Mean weight gain velocity in underweight and/or malnourished children with cyproheptadine hydrochloride.

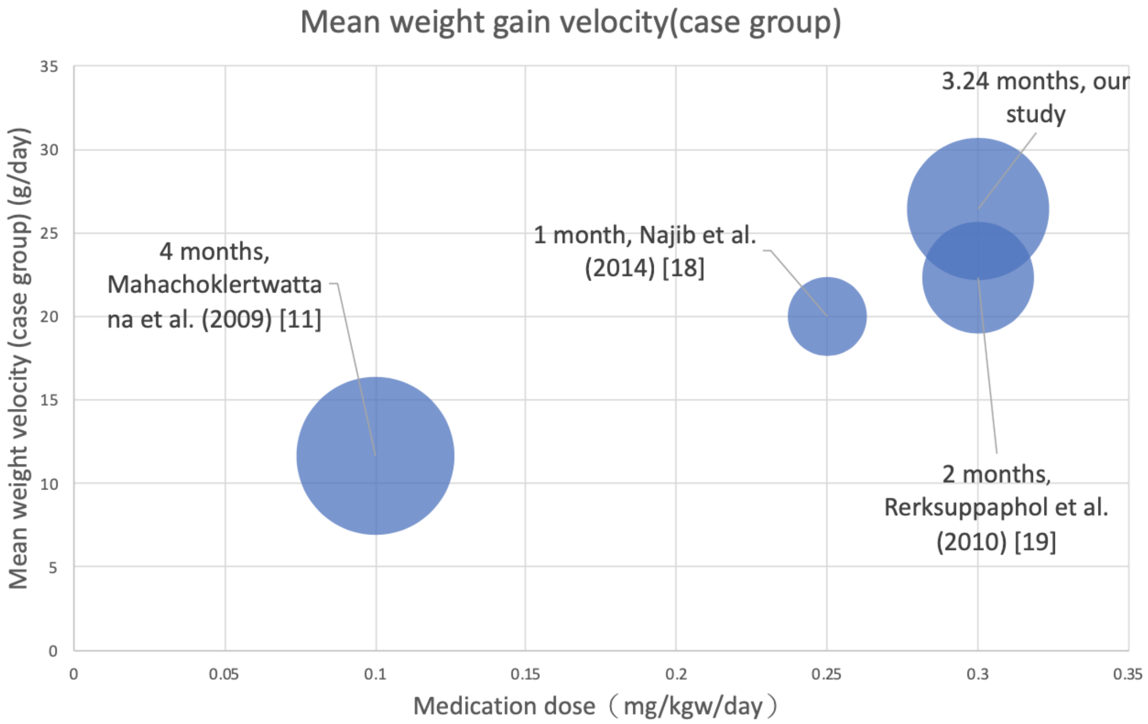

The bubble size is in proportion to medication duration.
