# Supplementary material for: Effects of cyproheptadine on body weight gain in children with nonorganic failure to thrive in Taiwan: A hospital-based retrospective study
Source: PLoS One. 2021 Oct 19;16(10):e0258731. doi: 10.1371/journal.pone.0258731 (PMC8525746; doi:10.1371/journal.pone.0258731)
Supplement: Attachment — Submitted filename: Response_20210618.pdf [file pone.0258731.s003.pdf]

Manuscript Number: PONE-D-21-05718R1

Manuscript Title: Effects of cyproheptadine on body weight gain in children with nonorganic failure to thrive in Taiwan: a hospital-based retrospective study

Responses to the reviewers

Reviewer #1:

Lin, et al. submit a manuscript reporting a retrospective review of 788 children seen in the China Medical University Hospital for failure to thrive between 2007 and 2016. Fifty children received cyproheptadine in an attempt to improve weight gain, while the remaining 738 were untreated. The investigators compared measures of height, weight, and body mass index at baseline. Standardized measures adjusting for age showed that there was no difference between the groups at baseline. Based on modeling, the investigators identified a linear relationship between the duration of cyproheptadine treatment and measures of weight and BMI.

Specific comments:

1. Line 37: It is unclear what the authors mean by "%BH, %BW, and %BMI (50th percentile for age and sex)" It does not appear to mean percentiles for height, weight, and BMI. Does it mean the percentage of the 50th percentile for these measures? If so, this is an unusual way to express the data. The authors should analyze their data in terms of percentiles or SD scores.

> **Response:** We apologize for the confusion. The patient values for height, weight, and BMI were adjusted for age using the values in the growth charts for Taiwanese children as the reference. Each value for each patient was matched to the median chart value for a child of the same age and sex as the patient. The patient value was then divided by the corresponding chart value, and the product was multiplied by 100. The resulting percentages are referred to as %BH, %BW, and %BMI. Smaller percentages indicate lower height, weights, and BMIs and greater divergence from the standard values. Since height and weight are obviously affected by race and region, we used growth charts specifically compiled for Taiwanese children.[1] We were unable to obtain the original data and thus could not calculate SD scores. The above information is now provided in the manuscript (Lines 111-118).
>
> Reference:
>
> [1] Chen W, Chang M-H. New growth charts for Taiwanese children and adolescents based on World Health Organization standards and health-related physical fitness. Pediatrics & Neonatology. 2010;51(2):69-79.

2. Lines 39-40: It is unclear whether the authors are referring to measures obtained at baseline or at follow up.

Response: We are referring to the follow-up period. To clarify this point, we revised the text as follows: "No statistically significant difference in the median age-adjusted value was noted between the T-group and NT-group during the follow-up period." (Lines 37-38)

3. Lines 45 and 202: What is meant by "we determined by consensus that CH was helpful…"

Response: We meant to say that our results showed that cyproheptadine hydrochloride (CH) helped children gain weight, which is consistent with previous results. To better express this point, we revised the text as follows: "Our findings underscored the positive association between CH and weight gain among prepubertal children." (Lines 40-42 and 183-184)

4. Line 59: Recommend "…children hospitalized with FTT have nonorganic etiologies…"

Response: We appreciate your suggestion and have revised the text as follows: "More than 86% of FTT cases in hospitalized children have nonorganic etiologies, and this percentage is likely to be higher in the outpatient setting." (Lines 55-57)

5. Lines 68-70 and 70-72: These two passages are redundant

Response: In accordance with your suggestion, we deleted "In particular, cyproheptadine, an appetite stimulant, has been reported to beneficially improve weight gain and linear growth [11, 15, 16]."

6. Various places in the manuscript: Reference is made to "prepuberty children." This should be "prepubertal children."

Response: We thank you for pointing this out and have replaced "prepuberty children" with "prepubertal children" throughout the manuscript. (Lines 42, 72, 76, and 193)

7. Various places in the manuscript: Cyproheptadine is abbreviated to CH, but this is not done consistently.

Response: We thank you for bringing this to our attention. After introducing the abbreviation for cyproheptadine hydrochloride (CH), we now use it consistently throughout the main text.

8. Lines 142-145: The fact that the majority of patients in the NT group only had two visits to the clinic while the T group visited 5 or more times is a confounder because the increased medical attention and focus on weight may have altered feeding practices.

Response: We agree with your comment. The results of a given visit may be dependent on those of the previous visit. In addition, in our study, the measurements for each subject were performed at irregularly spaced time points. Thus, we included the variable time as a fixed variable and modeled the data using a first-order autoregressive structure. This is now noted in the statistical analysis section of the Methods as follows: "The effects of medication days on the relative percentages of height, weight, and BMI were analyzed using linear mixed models with a first-order autoregressive structure, taking time, age, sex, and treatment group into account." (Lines 137–139) The difference in the number of patients is listed as a study limitation (Lines 119-121).

9. Table 2: What is "standard height/weight/BMI?"

Response: We thank you for your question. Standard height/weight/BMI refers to the values for these parameters at the 50th percentile (i.e. the median values) for a child of a given age and sex as derived from the growth charts for Taiwanese children and adolescents, which are based on the World Health Organization and health-related physical fitness standards. We defined this term in the Table 2 legend as follows: "The standard values for height, weight, and BMI were derived from the growth charts for Taiwanese boys and girls at ages corresponding to those of the patients. The chart values at the 50th percentile were used." (as Table 2)

10. Line 203: I think the authors mean 0.3 mg/kg daily.

Response: You are correct, and we have amended the wording accordingly. (Line 184)

11. In addition to the unusual expression of relative body measures, the authors do not discuss the changes in body weight in individual patients. The best approach would be to compare the change in height, weight, and BMI percentiles or SD scores in the T group vs. the NT group. It is not clear why they did not do this.

Response: We appreciate your comment. Comparing the changes in the height, weight, and BMI percentiles or in the SD scores between the groups would provide valuable information. However, the direct body measurements differed significantly between the T- and NT-groups at the first visit (Table 2). Hence, we did not and should not use these measurements for comparisons. Instead, we normalized the patient values to the corresponding age- and sex-matched values at the 50th percentile in the growth charts for Taiwanese children and adolescents.

12. It is not clear what this study adds to an already fairly extensive literature.

Response: We thank you for raising this important issue. Although epidemiology has shown that FTT is likely to occur in prepubertal children, the data are limited. In addition, the CH dosage and treatment length varied among the previous studies (e.g., from 0.1 to 0.3 mg/kg/day and 1 to 4 months, respectively). We found that the effects of CH are dose- and time-dependent. The weight gain (26.45 g/day) achieved in our study using 0.3 mg/kg/day was greater than that reported in previous studies using the same dosage, as was the speed with which normal weight was attained. Hence, our study shows that CH (0.3 mg/kg/day) efficiently promotes weight gain in prepubertal children with non-organic FTT. (Lines 183-187 & 241-248)

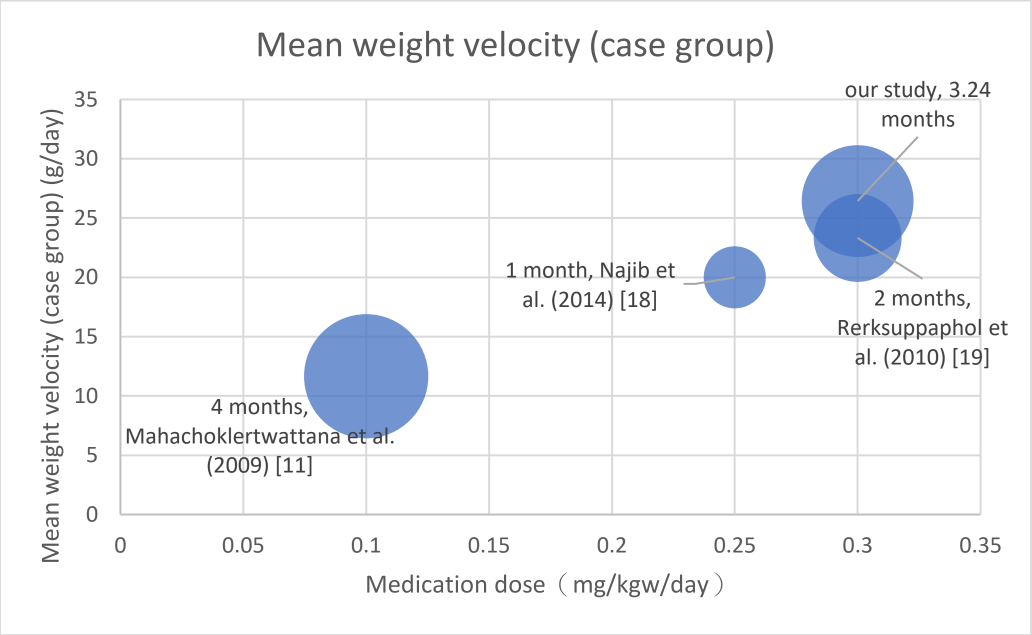

* The bubble size is in proportion to medication duration.

13. Additional data would be informative, including the dose used, duration of treatment, and pubertal status.

Response: In accordance with your recommendation, we now note the following in the revised manuscript:

- All patients received CH at 0.3 mg/kg daily for at least 14 days. (Lines 100)
- All children in our study were prepubertal (boys aged 3–11 years and girls aged 3–10 years at the first visit). (Lines 91)
  We also noted that “children <3 years-old at the first visit were excluded owing to the difficulty in distinguishing organic FTT from nonorganic FTT in this age group according to a recent report [23].” (Lines 96-97)
- The duration of the treatment ranged from at least 14 days to 532 days (mean: 97.22 days). (Table S1).

Reviewer #2:

The manuscript by Yi-Chun Lin and colleagues describes a retrospective cohort study looking at the effect of cyproheptadine on growth parameters in pre-pubertal children with non-organic failure to thrive. They showed that the group treated with cyproheptadine had statistically significant association with medication duration and %BW and %BMI. They were able to include a large number of patients compared to prior studies, but were limited due to its retrospective nature and multiple possible confounders.

Major comments/suggestions/questions:

1. Were the investigators able to gather information about prior behavioral or environmental interventions as well as nutritional supplements? Since these are common first line treatments it would be interesting to see if these patients had previously or concurrently used these treatments.

> **Response:** We appreciate your suggestion. In addition to prescribing CH, the doctors at our hospital provide FTT children and their parents with information regarding appropriate nutrition and feeding skills (behavioral interventions) and suitable dining environments, atmospheres, and appliances (environmental interventions) as the first-line treatment. Unfortunately, we were unable to assess the effects of interventions or of nutritional supplements on the study parameters owing to incomplete data in the medical records. However, all children with non-organic FTT in our study most likely received the same health education as the first-line treatment. Lack of assessment of prior health education and nutritional supplementation are cited in the revised manuscript as a study limitation. (Lines 258-263)

2. Since there have been many randomized control trials, it should be emphasized how this single center retrospective review adds to the known body of literature. One aspect that could be emphasized is the larger sample size. Another could be the real-world use of cyprohepatidine.

> **Response:** We thank you for this helpful suggestion. Accordingly, we have discussed the strengths of our single-center study in the discussion as follows: “CMUCHMC, the site of our study, is not only one of the first National Children’s Hospitals recognized by the Ministry of Health and Welfare in Taiwan but also the largest children’s hospital in central Taiwan. It receives a large number of visits (e.g., 179,832 clinic visits in 2016) and is very representative of the demography in central Taiwan. Therefore, the strengths of this study included its large sample size and real-world use of CH. Additionally, the longitudinal dataset allowed us to assess the effects of CH over time in a diverse group of patients. Lastly, the weight gain (26.45 g/day) achieved in our study using 0.3 mg/kg/day was greater than that reported in previous studies, as was the speed with which normal weight was attained.” (Lines 241-248)

3. Table 1 – The title could be made more clear with listing the number of visits as opposed to "visiting status" and visit 1, etc

Response: In accordance with your recommendation, column 1 in Table 1 is now titled “Number of visits” and the visits are listed as “1, 2, etc.”

4. Z scores for weight and height and BMI for age should be used and compared between groups. This is the standard to assess nutritional status in children. There is not much utility in comparing weights and heights without adjusting for age and sex. Also, it would great to include mid-parental height and accounted for it if possible.

Response: We agree with your assessment. However, we were unable to obtain the original data needed for the calculation of Z scores. The weights, heights, and BMIs in our study were adjusted for age and sex using the median values in the growth charts for Taiwanese children and adolescents as the reference.

5. Table 3 and 4 are confusing and require more explanation in the results section. There appears to be a negative association between treatment vs control on % BMI in table 3 based on how it is displayed. Could these results be better shown with a graph?

Response: We apologize for the confusion. In Table 3, the linear mixed-model analysis showed that the T-group had a more negative weight trend than did the NT-group, which is consistent with clinical observations. Patients with severe FTT will usually ask for a rigorous treatment (e.g., drug administration or surgery) rather than observation and tracking. The results in Table 3 also show a positive linear relationship between %BW and %BMI and between both %BW and %BMI and medication duration. Therefore, we stated that CH helps children gain weight.

6. The results section mentions an “equivalent weight gain of 26.43g/day,” but it is unclear how that number was obtained. Please explain how that was calculated.

Response: We gladly explain our calculations.

We first calculated the mean sex-adjusted standard weight for the T-group as follows:

$$21.7 \times 362 + 22.0 \times 426/788 = 21.86$$

where 21.7 is the mean weight of the girls in T-group, 362 is the number of girls in the T-group, 22.0 is the mean weight of boys in the T-group, 426 is the number of boys in the T-group, and 788 is the total number of girls and boys in the T-group. All values were derived from Table 2.

We then calculated the weight gain per day as follows:

$$0.121 \times 21.86\, kg = 0.02645\, kg/day\ (26.45 g/day)$$

where 0.121 (see Table 4) is the daily weight gain unit and 21.86 is the sex-adjusted standard weight in kg.

Hence, each additional day of medication time increases weight by 26.45. In the original manuscript, we did not adjust the standard weight for sex (as was done in the revised manuscript); this resulted in the previous value of 26.43 g/day.

These formulas have been added to the text (Lines 169-176)

Table 2. (abridged)

**Variable**	**ALL** *N* **= 788**	**NT-group** *N* **= 738**	**T-group** *N* **= 50**	***P* value**
**Standard value for girls (*N* = 362)[a]**				
**Weight**	23.2 ± 5.7	23.3 ± 5.7	21.7 ± 6.9	0.2498
**Standard value for boys (*N* = 426)[a]**				
**Weight**	25.3 ± 6.9	25.6 ± 6.9	22.0 ± 6.4	0.0051**

7. It would be beneficial to include the dose and duration of cyproheptadine use in the Methods section or as part of the results.

Response: The dose of cyproheptadine was 0.3 mg/kg daily. The duration of the treatment was 97.22 days (3.24 months), range: >14 to 532 days. We have added this information to the manuscript (Lines 100, and Table S1)

8. Were side effects reported and could they be included in this manuscript? Regardless of that data being available, there should also be a discussion of potential side effects of using cyproheptadine in the discussion section.

Response: As requested, we now discuss the potential side effects of CH as follows: “As summarized in a retrospective review, the side effects of CH are fairly benign in children with feeding problems [7]. Reported side effects such as tachycardia, constipation, diarrhea, irritability, and sleepiness are resolved by decreasing the dosage of CH or discontinuing the treatment [7, 27, 28]. Future research exploring the potential intermediary role of side effects on the association between CH treatment and body weight is suggested.” (Lines 214-219)

9. In the discussion it is mentioned that the higher the CH dose the child received the more weight velocity was attained, but the comparison between this real-world study and the RCTs cannot lead to this conclusion. It might be inferred from the comparison but unless a full study is done, the conclusion the authors draw cannot be made. This should be changed in the discussion.

Response: We thank you for raising this point. In the three RCTs in Table S1, the mean daily weight gain for 0.1, 025, and 0.3 mg/kg/day CH was 11.66, 20, and 22.32 g/day respectively. In our study, which used 0.3 mg/kg/day, it was 26. 45. Hence, increasing the CH dosage increased the weight gain velocity. The relationship between CH dosage and weight gain velocity is illustrated in the table below.

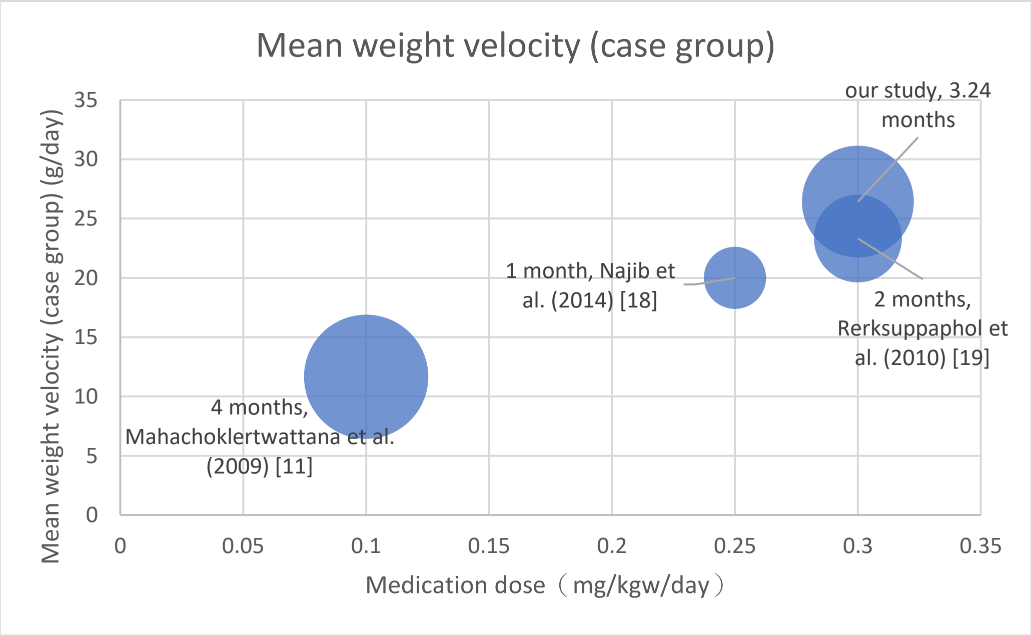

* The bubble size is in proportion to medication duration.

To clarify this point, we now state the following: “Low doses of CH (0.1–0.3 mg/kg/day) have been shown to have a pronounced effect on weight gain in children (Table S1). Increasing the CH dose from 0.1 to 0.3 mg/kg/day increased mean daily weight gain from 11.66 to 22.32 g/day and accelerated the attainment of normal weight in children in random control trials. The CH dosage for prepubertal children with non-organic FTT was the same in our study and that by

Rerksuppaphol et al. [19]; however, the mean daily weight gain in our study was higher (26.45 g/day vs. 22.32 g/day).

10. Table 5 in general seems unnecessary and could be moved to being a supplemental table.

> **Response:** Table 5 is now included as supplementary information in accordance with your recommendation.

Minor comments:
11. De-identified data should be made available upon request

> **Response:** In regard to your comment, we contacted the data management center and medical ethics committee at our hospital. We were informed that the dataset used in our study was only available to the members of our hospital and affiliated institutions.

12. The increased number of clinic visits seen between the treatment groups should also be included as a possible confounder. The more frequent visits might indicate that the parents are more likely to be motivated to try an intervention and use other therapies.

> **Response:** To control for the difference in the number of visits between the T- and NT-groups, we incorporated the number of visits into the time variable in linear mixed models, as shown in Table 4. This is noted in the Methods section (Lines 120-121), and the difference in the number of visits was listed as a study limitation.
> (Lines 270-272)

Reviewer #3

This retrospective study examines the effects of cyproheptadine on weight gain and vertical growth in a small cohort of 50 children ages 3-11 diagnosed with nonorganic failure to thrive treated with cyproheptadine between 2007-2017 in an academic tertiary care center in Taiwan. These children are compared to a fairly large untreated cohort. Using "Linear Mixed Modeling", the authors conclude that cyproheptadine improved %BMI and %BW in children with nonorganic failure to thrive. I think this has potential to support published data on the benefits of cyproheptadine for this specific age group.

Major Critiques:

1. This study does not add significant new contributions to the body of literature, as numerous published articles (including a systematic review and two prospective trials appropriately cited in the text) highlight the beneficial effects of cyproheptadine on growth. The authors cite differences in dosing and population standardization in prior studies as reasons to publish this data, but the cohort has significant age variation, and the authors do not clearly delineate if/what variation in cyproheptadine dosing was used in the patients in their cohort. Of note, suggestions for dosing exist for cyproheptadine as an appetite stimulant in medication dosing reference resources such as UpToDate.

> **Response:** Our study used a longitudinal dataset to address changes in weight in low-dose CH-treated patients over time, which have not been examined previously. For this reason, we believe that our study is of value. To highlight the strengths of our study, we have added the following to the Discussion: "CMUCHMC, the site of our study, is not only one of the first National Children's Hospitals recognized by the Ministry of Health and Welfare in Taiwan but also the largest children's hospital in central Taiwan. It receives a large number of visits (e.g., 179,832 clinic visits in 2016) and is very representative of the demography in central Taiwan. Therefore, the strengths of this study included its large sample size and real-world use of CH. Additionally, the longitudinal dataset allowed us to assess the effects of CH over time in a diverse group of patients. Lastly, the weight gain (26.45 g/day) achieved in our study using 0.3 mg/kg/day was greater than that reported in previous studies, as was the speed with which normal weight was attained." (Lines 241-248)

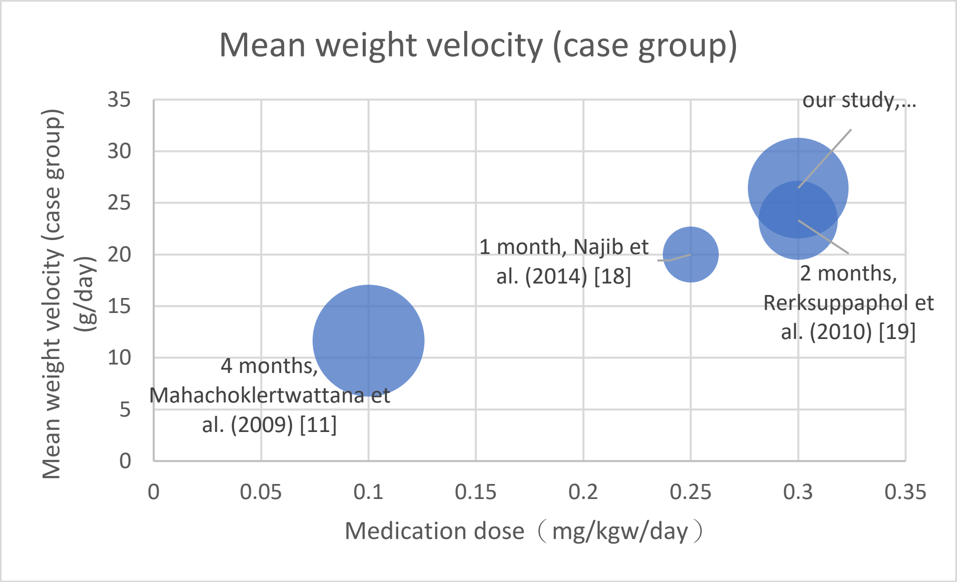

* The bubble size is in proportion to medication duration.

Minor Critiques:

Few minor grammatical errors should be addressed throughout the manuscript

> **Response:** We thank you for pointing this out. The revised manuscript has been proofread by native English speakers.

1. PAGE 8, line 104: Recommend defining what qualifies as a "long-term medicines". Would this be other appetite stimulants? Stimulant medications for ADHD that might suppress appetite? Would a multivitamin or other medication such as polyethylene glycol that would most likely be unrelated to growth exclude a patient?

> **Response:** We have modified our wording to make it clear that we are referring to long-term (> 3 months) use of appetite stimulants (including traditional Chinese medicines) other than CH (Line 99-100)

2. PAGE 9, lines 117-119: Recommend including what specific statistical tests were used to compare treatment groups (ie t-test is cited in Table 2). Additional description of how "Linear Mixed Models" were used for statistical analysis is extremely important, as the conclusions are completely based on this analysis.

> **Response:** We appreciate these suggestions. We used the t-test to compare continuous variables between the control and treatment groups; this is now noted in the Table 2 legend and in the statistical analysis section of the Methods. (Lines 118-119)

For the linear mixed models, we used the variable time as a fixed variable and modeled the data using a first-order autoregressive structure. This was done because the number of clinic visits differed between T- and NT-groups and also among the subjects, and the results of a given visit may be dependent on those of the previous visit. In addition, the measurements for each subject were performed at irregularly spaced time points. We have revised the text in the statistical analysis section of the Methods as follows: "The effects of medication days on the relative percentages of height, weight, and BMI were analyzed using linear mixed models with a first-order autoregressive structure, taking time, age, sex, and group into account." (Lines 119-122)

3. PAGE 13, Table 2: Very "busy" table. Could simplify to facilitate interpretation by excluding "N" columns and instead include the "N" data below column or row headings, and combine the Mean SD columns into Mean (SD)

Response: We thank the reviewer for these suggestions and have modified Table 2 accordingly.

4. PAGE 14, Table 3: This table does not clearly communicate desire results. Interpreting results in this format is not intuitive. Unclear what "Intercept" and "Time" refer to. Better represented graphically?

Response: We apologize for the confusion. In a linear mixed model, the intercept is calculated as $\beta_1 x_1 + \beta_2 x_2 + \beta_3 x_3 + \beta_4 x_4 + \beta_5 x_5 + \beta_6 x_6 + \gamma$), where γ is the intercept. Time is one of the variables in the model.

The height and weight of children increase over time and were measured at multiple times in our study. Moreover, owing to differences in patient characteristics (e.g., age at FTT onset, year of consultation, duration of treatment, and follow-up time), we were unable to directly compare changes in height, weight, and BMI in terms of percentiles or SD scores between the T- vs. NT-group. Therefore, we used a linear mixed model, which we believe was the most suitable tool for our analysis. A linear mixed model is mainly used in longitudinal studies, and its variables are repeatedly measured (for example: a patient visits a hospital three times, and the record of each visit or biological indicators). The variables are not independent of each other between each measurement.

5. PAGE 15, Table 4: Similarly, a confusing table. Unclear what "Intercept" and "Time" refer to. Better represented graphically?

Response: Please see the response to comment 4.

6. PAGE 15, Line 202-206: It is unclear where the outcomes stated in the main findings (0.3 mg of CH x14 days) is associated with increased BW gain and BMI are represented in the figures/tables.

Response: For clarification, we revised the text as follows: "Our findings reveal a positive association between CH intake and weight gain among prepubertal children. We found that continuous intake of CH (0.3 mg/kg/day) for at least 14 days (mean: 3.24 months) increased the weight gain velocity and BMI in a linear fashion among prepubertal children (age range: 3–11 years) with mild-to-moderate wasting (60–74% of the median weight standard) but without medical conditions." Lines 183-187)

7. PAGE 16, Line 211-213: Discussion about dose of cyproheptadine for appetite stimulation and subsequent reference to Table 5 is disjointed. Strongly recommend rewording.

Response: This section of the manuscript has been revised for clarity. We hope it is now acceptable to you

8. PAGE 16, Table 5: The dosing of cyproheptadine should be introduced in the "Methods" section. The first mention of the dose of cyproheptadine occurs in the "Discussion section"

Response: As requested, we have introduced the cyproheptadine dosage in the Methods section. (Lines 33 & 100)
It is now also noted at the beginning of the Results section (Tale 3 &Table 4).

9. Figure 1: This figure is not cited in the text. The figure shows patients treated with traditional Chinese medicine were excluded, but this is not mentioned in the body of the text as one of the exclusion criteria

Response: We apologize for this oversight; Figure 1 is now cited (Line 137). Information regarding the exclusion of patients treated with traditional Chinese medicines is included in the revised manuscript. In the list of the exclusion criteria, we state the following: "long-term (>3 months) use of appetite stimulants other than CH including traditional Chinese medicines." (Lines 99-100)
