# Supplementary material for: Effects of cyproheptadine on body weight gain in children with nonorganic failure to thrive in Taiwan: A hospital-based retrospective study
Source: PLoS One. 2021 Oct 19;16(10):e0258731. doi: 10.1371/journal.pone.0258731 (PMC8525746; doi:10.1371/journal.pone.0258731)
Supplement: Attachment — Submitted filename: Response_20210812.pdf [file pone.0258731.s004.pdf]

Manuscript Number: PONE-D-21-05718R2

Manuscript Title: Effects of cyproheptadine on body weight gain in children with nonorganic failure to thrive in Taiwan: a hospital-based retrospective study

---

Responses to the reviewers

Reviewer #1:

Specific comments:

1. Table 4 indicates that medication duration is positively associated with gains in %BW and %BMI. Could there be a selection bias here that explains this relationship? Those who had a good response might be more likely to continue medication use, while those who did not respond might abandon the treatment early.

   Response: Thank you very much for reviewing our revision. We appreciate your comments on this issue. Selection bias may occur in this study, but we believe it has little impact on our results. It was because that there are no optimal recommendations for the dosage and medication duration of cyproheptadine hydrochloride (CH) for children with failure to thrive. As a result, clinicians did not know how much and how long it would take CH to be effective or when the drug should be discontinued. In this study, clinicians stopped prescribing CH to patients because patients did not return to the clinics, not simply because the weight gain was unsatisfactory. Most of the reasons why the patients did not return to the clinics included a poor response to drugs, side effects, and low compliance. Although the exact reason cannot be determined since this retrospective study had no data on the reasons for drop-outs, the risk of bias resulted from weight gain unsatisfactory was low.

2. Lines 211-213: The inability to assess the relationship beyond 4 months of treatment should be stated in the Results section as well.

   Response: We appreciate your suggestion. We agree that 4-month should be a limitation when interpreting our result and have added the following text in the revision:

   > "Nevertheless, we were unable to assess whether the linear relationship between weight gain velocity and medication duration could extend beyond 4 months. It was because the medication duration in our study varied widely, ranging from 14 days to 532 days, and the number of subjects was insufficient for stratified analysis." (lines 178-181)

Reviewer #2:

Major comments/suggestions:

1. It would be more useful to have a mid-parental height as opposed to seeing the maternal and paternal heights separately.

   Response: Thank you very much for reviewing our revision. We appreciate your comments and suggestions. We calculated the mid-parental height by adding 6.5 cm to the average of both parents' heights for boys or subtracting 6.5 cm from the average of both parents' heights for girls. We found that there was no significance in maternal and paternal heights separately between T-group and NT-group (*P* = 0.1616). The results have been added in **Table 2**.

2. Please include units for variables in Table 2. For example, in rows for standard value for girls/boys should include (cm, kg) and for age-adjusted values should include (%). It is described in footnote, but should also be included in the table.

   Response: We agree with your suggestions and have added units for variables in **Table 2**, including standard height in cm, standard weight in kg, age-adjusted values in %, and mid-parental height in cm.

3. Tables 3 and 4 are difficult to interpret alone without the explanation found in the text. Please display this information graphically in addition to showing the tables. This will make the data easier to interpret and will be visually more impactful.

   Response: We used a linear mixed model for repeat measurements. The coefficient estimates in Table 3 were estimated based on the following equation of a linear mixed model, whereas Table 4 followed a similar model but excluding $\beta_2 \times \mathrm{group}$.

$$\begin{aligned}\text{age} - \text{ and sex} - \text{adjusted outcome variables} \\ = \text{intercept} + \beta_1 \times \text{time} + \beta_2 \times \text{group} + \beta_1 \times \text{sex} + \beta_1 \times \text{age} \\ + \beta_1 \times \text{medication duration}\end{aligned}$$

   Both the main predictor (i.e., medication duration) and outcome variables were continuous variables. In addition, we didn't transfer or categorize our data. Therefore, the effect of the predictor on outcome variables was presented in estimated coefficients. It is unnecessary and impractical to display a single coefficient for a predictor in graphics. We still appreciate your kind suggestions.

Minor comments:

4. Line 99 is missing closed parenthesis

   Response: We appreciate your remark and have added the closed parenthesis. (lines 99)

5. Line 101 should say “choices made by patients and their parents in consultation with their physicians”.

   Response: We appreciate your suggestion and have revised the text as follows: “Because our study was retrospective, the allocation of treatment modalities was based on previous choices made by patients and their parents in consultation with their physicians.” (lines 100-101)

6. The formula for the calculation of average standard weight should be included in the methods and not the results section.

   Response: We appreciate your remark. Our description regarding the calculation of average standard weight is in the section Statistical analysis, which is under the Methods section. (lines 109-117)

7. It would be nice if the new figure comparing this study with the others was included as a supplemental figure and referenced in the discussion section. It seems a shame for this figure to be only for the reviewers.

   Response: Thank you very much for your suggestion. We have included this figure in our supporting information, i.e., Figure S1. (line 200 and page 29)
